# Deterministic distribution of four-photon Dicke state over an arbitrary collective-noise channel with cross-Kerr nonlinearity

**DOI:** 10.1038/srep29853

**Published:** 2016-07-14

**Authors:** Mei-Yu Wang, Feng-Li Yan, Ting Gao

**Affiliations:** 1 College of Physics Science and Information Engineering, Hebei Normal University, Shijiazhuang 050024, China; 2 College of Mathematics and Information Science, Hebei Normal University, Shijiazhuang 050024, China

## Abstract

We present two deterministic quantum entanglement distribution protocols for a four-photon Dicke polarization entangled state resorting to the frequency and spatial degrees of freedom, which are immune to an arbitrary collective-noise channel. Both of the protocols adopt the X homodyne measurement based on the cross-Kerr nonlinearity to complete the task of the single-photon detection with nearly unit probability in principle. After the four receivers share the photons, they add some local unitary operations to obtain a standard four-photon Dicke polarization entangled state.

Entanglement^1^,^2^,^3^ plays an important role in quantum information processing, mainly including quantum computation^4^ and quantum communication. It is the information carrier in some interesting branches of quantum communication, such as quantum key distribution^5^, quantum secret sharing^6^,^7^,^8^, quantum secure direct communication^9^,^10^,^11^, teleportation^12^, quantum dense coding^13^,^14^, and so on. In all of the above applications, two- or multi-qubit maximally entangled states must be shared as quantum channels by the parties at different locations. As photons are ideal carriers for long-distance transmission of quantum states, people always choose their entangled states in the polarization degree of freedom (DOF) to fulfill these tasks discussed previously. However, during a practical transmission, the polarization DOF of photons is easily influenced by the thermal fluctuation, vibration, and the imperfection of an optical fiber. That is, they suffer from the channel noise inevitably whether they are single photons or multi-qubit entangled photons. Thus, various error correction and error-rejection processes are proposed. A method of distilling a maximally entangled state is entanglement purification, which can be used to decrease the influence of the channel noise and will be an efficient method in the case that the distance is not so long. For instance, Bennett *et al*.^15^ proposed an original entanglement purification protocol (EPP) for purifying a Werner state based on quantum controlled-NOT gates in 1996. In 2001, Pan *et al*.^16^ proposed an EPP based on linear optics, without resorting to controlled-NOT gates, which is feasible in experiment. Sheng *et al*.^17^ proposed an EPP based on cross-Kerr nonlinearity. However, entanglement purfication is essentially used to achieve a subset of maximally entangled states from less-entangled ones after infinite operations. All conventional EPPs cannot get perfect maximally entangled photons by far as they work probabilistically in principle. Thus, the faithful distribution of maximally pure entangled states between different and distant locations is valuable for the realization of long-distance quantum communication.

The polarization entanglement of photons^18^,^19^,^20^ is easily disturbed by the noise in quantum channel, so it is not an elegant way to directly transmit the polarization entanglement of photons over a noisy channel. Recently, some other DOFs attract much attention, such as the frequency DOF, the spatial mode, orbital angular momentum and so on. For example, since the bit-flip error and phase-flip error can be corrected correspondingly as the frequency entanglement state is a maximally entangled pure state, and the frequency entanglement does not easily suffer from the channel noise in principle^21^, Sheng and Deng^22^ proposed an Einstein-Podolsky-Rosen pair distribution protocol over an arbitrary collective-noise channel exploiting conversion between polarization and frequency modes. The protocol can be generalized to the distribution of *n*-qubit (*n* > 2) Greenberger-Horne-Zeilinger (GHZ) state. This protocol is very important because multi-qubit entangled states have many advantages over the two-qubit entangled states in quantum information. Since then, in 2011, Lu *et al*.^23^ proposed an efficient W polarization entangled state distribution protocol over an arbitrary collective-noise channel with the help of the cross-Kerr nonlinearity. In 2013, Dong *et al*.^24^ rendered a perfect entanglement distribution protocol of a four-photon *χ*-type polarization-entangled state exploiting spatial DOF to depress the effect of collective noise. For four-qubit entangled states, Verstraete *et al*.^25^ showed that there are nine families of states under stochastic local operations and classical communication, such as the above mentioned four-qubit *χ*-type states, four-qubit GHZ states, four-qubit W states, and four-qubit cluster states. However, other nonequivalent classes of quantum states with interesting symmetries exist. For example, a novel four-qubit entangled state 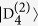
—the four-qubit Dicke state with two excitations that is symmetric under all permutations of qubits^26^, has the form as


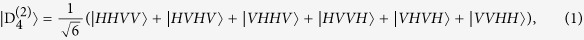


where *H* and *V* denote horizontal and vertical linear polarizations respectively. The four-photon state 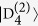, like |*W*〉_4_, is highly persistent against photon loss and projective measurements. In particular, Kiesel *et al*.^27^ showed that, in spite of the impossibility to transform a three photon GHZ type into a W state by local manipulation, both can be obtained via a projective measurement of the same photon in the state 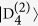. Dicke states constitute a particularly relevant class of highly entangled, they have interesting applications in quantum information processing tasks, such as 1 → (*N* − 1) telecloning or open-destination teleportation^28^,^29^ and quantum games^30^,^31^. Experimentally, high-fidelity Dicke states with small particle numbers have been created with photons^32^,^33^.

In this paper, we present two deterministic quantum entanglement distribution protocols for four-photon Dicke state in polarization over an arbitrary collective-noise channel with the help of the cross-Kerr nonlinearity. The two protocols exploit the frequency DOF and spatial DOF to against channel noise respectively, which are immune to an arbitrary collective-noise channel. Both of these two protocols adopt the X homodyne measurement based on the cross-Kerr nonlinearity to complete the task of the single-photon detection with nearly unit probability in principle. After the four receivers share the photons, they add some local unitary operations to obtain a standard four-photon Dicke polarization entangled state deterministically. We describe the explicit distribution scheme of the Dicke polarization entangled state with frequency entanglement in Section 2. The deterministic quantum entanglement distribution with spatial entanglement is shown in Section 3. Finally, the discussion and conclusion are presented in Section 4.

## Entanglement distribution of the Dicke state by frequency degree of freedom

For the sake of the clearness, let us first introduce the cross-Kerr nonlinearity, which was first used by Chuang and Yamamoto to realize the simple optical quantum computation^34^. The interaction Hamiltonian has the form 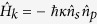, here 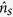 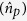 is the photon-number operators of the signal (probe) mode, and *κ* is the strength of the nonlinearity. If the signal field contains *n* photons and the probe field is in an initial coherent state with amplitude *α*, the cross-Kerr nonlinearity interaction causes the combined signal-probe system to evolve as follows:


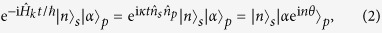


where *θ* = *κt* with *t* being the interaction time. It is easy to observe that the Fock state is unaffected by the interaction but the coherent state picks up a phase shift *nθ* directly proportional to the number of photons *n* in the signal mode. One can exactly obtain the information of photons in the Fock state but not destroy them by detecting the probe mode with a general homodyne-heterodyne measurement. The cross-Kerr nonlinearity between photons offers an ideal playground for quantum state engineering, and a number of applications have been studied, such as constructing nondestructive quantum nondemoliton detectors (QND)^35^,^36^, deterministic entanglement distillation^37^, logic-qubit entanglement^38^,^39^, generation of multiphoton entangled state^40^,^41^,^42^,^43^,^44^. In what follows, we explain the distribution process of the four-photon Dicke state over an arbitrary collective-noise channel with frequency DOF. On experiment, with present technology, the entanglement of photons in frequency DOF is not difficult to be prepared with spontaneous parametric down-conversion^45^,^46^. Frequency DOF has been used in a series of quantum information schemes because of its stability. We suppose that the center, say Susan wishes Alice, Bob, Charlie and David to share a polarization photon state 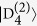 as described in Eq. (1). By means of method in refs 45,46 she prepares a four-photon Dicke state


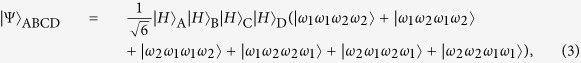


where, the notions |*ω*_1_*ω*_1_*ω*_2_*ω*_2_〉, |*ω*_1_*ω*_2_*ω*_1_*ω*_2_〉, 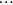, |*ω*_2_*ω*_2_*ω*_1_*ω*_1_〉 are six different frequency modes of the four photons. The subscripts A, B, C, and D mean that the four photons are distributed to Alice, Bob, Charlie and David, respectively. Suppose the collective noises in the four channels have the same form but different noise parameters which alter with time in principle, i.e.,


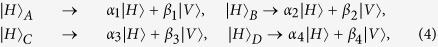


where |*α*_*i*_|^2^ + |*β*_*i*_|^2^ = 1 (*i* = 1, 2, 3, 4). The four-photon entangled state in Eq. (3) suffering from collective-noise channels is written as


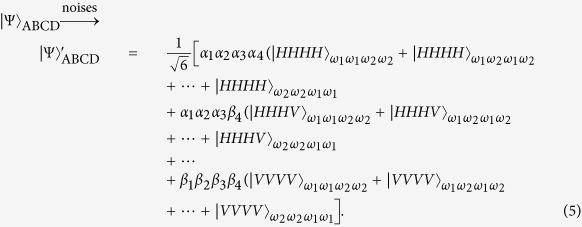


**Figure 1:**
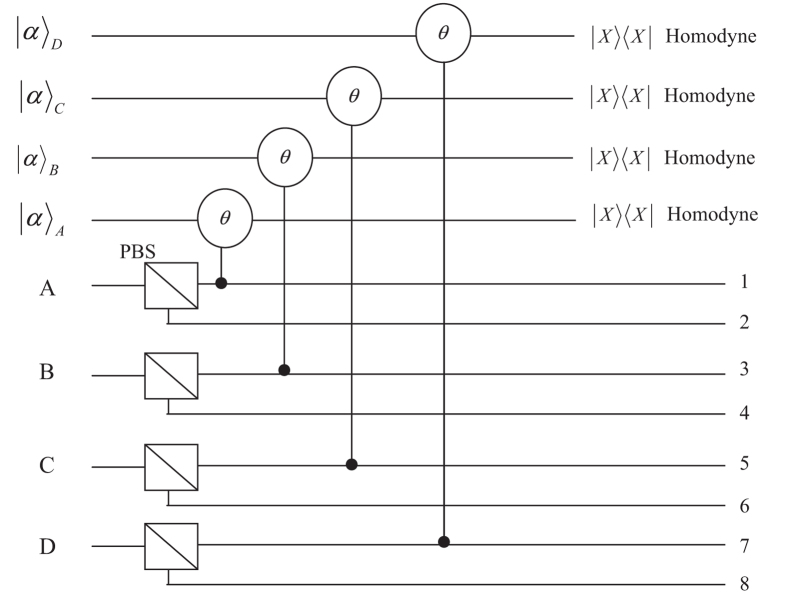
Schematic drawing of Dicke state distribution over a collective-noise channel with the help of the cross-Kerr nonlinearities. PBSs are polarization beam splitters. Cross-Kerr nonlinearities will cause the coherent beam to pick up a phase shift *θ* if there is a photon in the modes 1, 3, 5 and 7.

After the noise channels, the resulting photons will pass through the polarization beam splitters (PBSs) which transmit the horizontal polarization mode |*H*〉 and reflect the vertical polarization mode |*V*〉. When Alice, Bob, Charlie and David combine their photons and their coherent probe beams with cross-Kerr nonlinearity media (shown in Fig. 1), the state 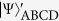 with the four coherent states evolves as


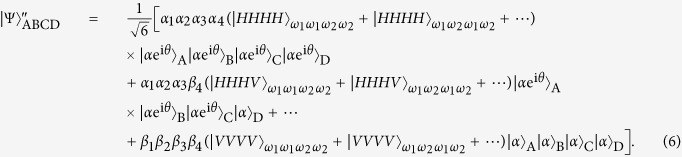


If a photon appears in the mode 1 (3, 5 or 7), the cross-Kerr nonlinearity puts a phase shift *θ* on the coherent state |*α*〉_A_ (|*α*〉_B_, |*α*〉_C_ or |*α*〉_D_). Meanwhile, if a photon appears in the mode 2 (4, 6 or 8), the coherent state |*α*〉_A_ (|*α*〉_B_, |*α*〉_C_ or |*α*〉_D_) picks up no phase shift. After the X homodyne measurements on their coherent beams independently, Alice, Bob, Charlie and David will get some different phase shifts, and the four photons will collapse into different states with different phase shifts. For example, if Alice, Bob, Charlie and David have the same phase shift *θ*, the four photons will collapse into the state


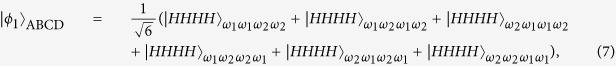


which appears at output modes 1357 with the probability of |*α*_1_*α*_2_*α*_3_*α*_4_|^2^. In a similar way, the other fifteen entangled states |*ϕ*_*i*_〉_ABCD_: 2 ≤ *i* ≤ 16 can be distinguished. Here


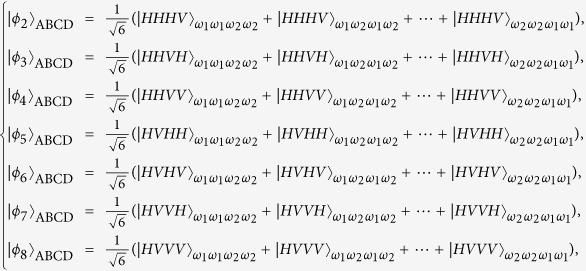



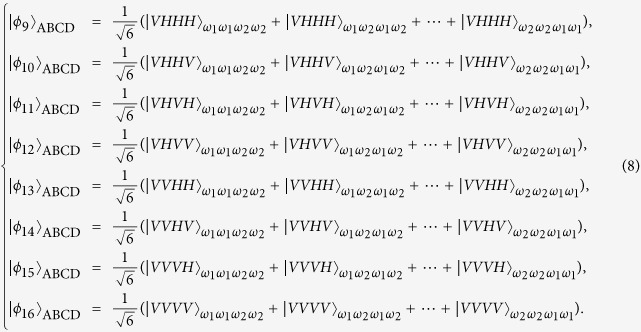


**Figure 2:**
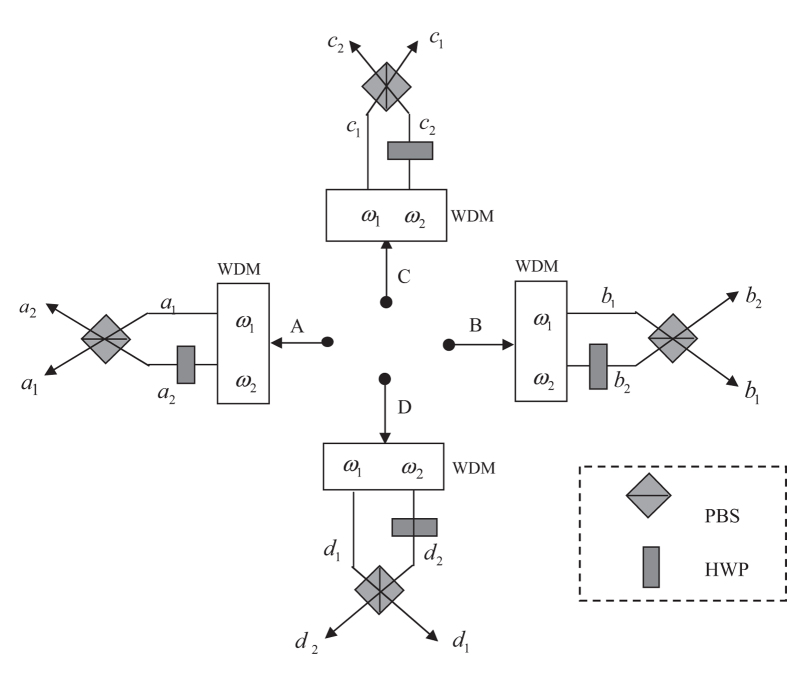
Schematic illustration of converting frequency entanglements to polarization entanglements. WDMs represent wavelength division multi-plexers, HWPs denote half-wave plates which realize the conversion between |*H*〉 and |*V*〉.

The next step of our Dicke state disrtibution protocol is to convert the frequency DOF entangled states {|*ϕ*_*i*_〉_ABCD_} to polarization DOF entangled ones. Without loss of generality, we take |*ϕ*_1_〉_ABCD_ as an example to describe the principle of the entanglement reconstructing process, shown in Fig. 2. Four polarization independent wavelength division multiplexers (WDMs) can be used to guide photons to different spatial modes according to their frequencies. That is, photons with the frequencies *ω*_1_ and *ω*_2_ will be guided to the corresponding spatial modes *a*_1_(*b*_1_, *c*_1_, *d*_1_) and *a*_2_(*b*_2_, *c*_2_, *d*_2_). Four half-wave plates (HWPs) are used to complete the transformation 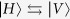 in suitable positions. After the four photons are coupled by the four PBSs, its state becomes


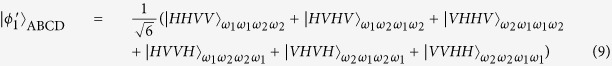


and will be in the output spatial modes *a*_1_, *b*_1_, *c*_1_ and *d*_1_. The remaining states in {|*ϕ*_*i*_〉_ABCD_ : 2 ≤ *i* ≤ 16} are analogical with it. Following the similar way, Alice, Bob, Charlie and David can obtain 16 maximally entangled states in the polarization and frequency degrees of freedom. Finally, four participants can erase the distinguishability for the frequency of their photons with the help of quantum frequency up-conversion^47^ and turn them into a standard polarization entangled Dicke state with local unitary operations. The collapsed states corresponding to the X homodyne measurement, together with the explicit output modes, the corresponding probabilities, and the corresponding local operations on photons can be seen in Table 1. The Dicke state distribution protocol has been successful and the success probability is 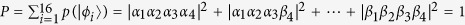. That is, this entanglement distribution process can be implemented with a unit probability in principle over collective-noise channels since it is independent of the noise parameters {*α*_1_, *β*_1_, *α*_2_, *β*_2_, *α*_3_, *β*_3_, *α*_4_, *β*_4_}.

## Entanglement distribution of the Dicke state by spatial degree of freedom

Besides the frequency DOF, the spatial DOF is also robust to the channel noise. As for the spatial entanglement, the bit-flip error does not exist and the phase-flip error can be eliminated by controlling the lengths of channels exactly, so the spatial DOF can also be used to create the entanglement in polarization DOF. In this section, we demonstrate another scheme for distribution of entanglement with the spatial entanglement.

**Figure 3:**
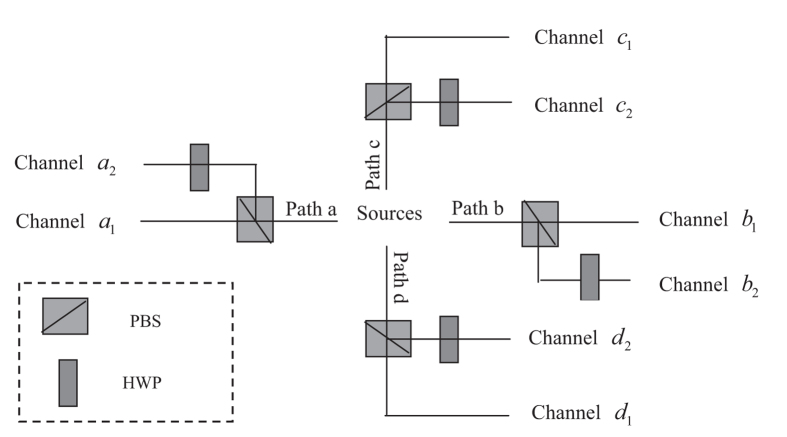
Schematic illustration of transmitting a four-photon Dicke polarization entangled state. The symbols ‘*a*, *b*, *c*, *d*’ denote photon paths, and the subscripts ‘1, 2’ denote the corresponding upper and lower channels, through which four photons of the entangled state are transmitted.

Suppose the center Susan has a polarization entangled Dicke state shown in Eq. (1), which can be generated by means of method in refs 32,33. First, she transforms the entangled mode of the Dicke state from the polarization DOF mode to the spatial DOF mode, by utilizing the combination of PBSs and HWPs, as shown in Fig. 3. Then the state to be transmitted in Susan’s is changed to


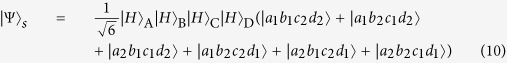


with local unitary operations. Here *a*, *b*, *c*, and *d* represent the four spatial modes of the entangled system. Subsequently, the four photons are transmitted through the collective-noise channels. Here we suppose channels *x*_1_ and *x*_2_ (*x* = *a*, *b*, *c*, *d*) are so close that the noise affected on photons are identical, which is given as


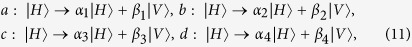


where |*α*_*i*_|^2^ + |*β*_*i*_|^2^ = 1 (*i* = 1, 2, 3, 4). The state denoted as Eq. (10) suffered from collective-noise channels is evolved to


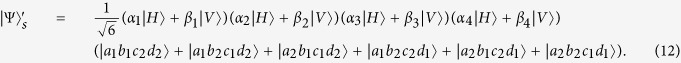


**Figure 4:**
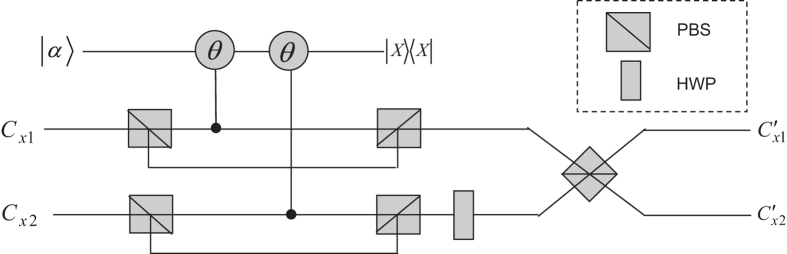
Schematic illustration of receiving a four-photon Dicke polarization entangled state. The symbol ‘*x*’ denotes photon paths (*a*, *b*, *c*, *d*). The composition of PBSs and X homodyne measurement determine the polarization part of the evolved state.

Finally, the four photons arrive at receivers Alice, Bob, Charlie and David. In the receiving process, every receiver introduces a probe beam with cross-Kerr nonlinearity media (shown in Fig. 4) and then performs the X homodyne measurement independently. As a result, the polarization part of the four-photon Dicke state collapses into |*HHHH*〉, |*HHHV*〉, 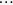 with the probabilities of |*α*_1_*α*_2_*α*_3_*α*_4_|^2^, |*α*_1_*α*_2_*α*_3_*β*_4_|^2^, 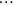. In the end, the four photons pass through the combination of HWP and PBSs, and the transmitted state can be transformed to the original Dicke state with some local unitary operations. All X measurement outcomes (polarization states), together with the explicit output and the corresponding local operations on photons can be seen in Table 2. The successful distribution of the four-photon polarization entangled Dicke state is confirmed and the total probability is *P* = |*α*_1_*α*_2_*α*_3_*α*_4_|^2^ + |*α*_1_*α*_2_*α*_3_*β*_4_|^2^ + 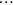 + |*β*_1_*β*_2_*β*_3_*β*_4_|^2^ = 1.

We can generalize the above scheme to the case for distribution of 2*n*-qubit system (*n* > 2) in a highly entangled and symmetric Dicke state over an arbitrary collective-noise channel. The highly symmetric Dicke state 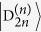 is given by


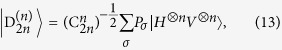


where 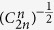 is a normalization factor with 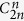 as binomial coefficient, and ∑_*σ*_ *P*_*σ*_(…) means the sum over all permutations of the photonic qubits. Through the setup shown in Fig. 3, the state to be transmitted is transformed into


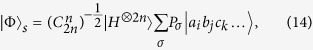


where *i*, *j*, *k*, … ∈ 1, 2. After passing through the collective-noise channels, the 2*n*-qubit system evolves as


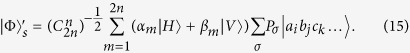


Similar to Fig. 4, every receiver use their QNDs with cross-Kerr nonlinearity to check the polarization part of the 2*n*-photon Dicke state, and then, the 2*n* photons pass through the combination of HWP and PBSs. Finally the transmitted state can be transformed to the original Dicke state 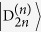 with some bit-flip operations on part of the 2*n* photons.

## Discussion and Conclusion

In the process of describing the principle of our protocols, we exploit the cross-Kerr nonlinearity interaction between photons and the coherent states. Although a lot of works have been studied in the area of cross-Kerr nonlinearities, we should acknowledge that it is still a quite controversial concept to have a clean cross-Kerr nonlinearity in the optical single-photon regime with present science and technology. In nature, cross-Kerr nonlinearity is extremely small and unsuitable for single photon interaction. Fortunately, it was suggested that the nonlinearity magnitude could be *θ* ~ 10^−2^ with the help of electromagnetically induced transparency^48^. Moreover, in the regime of weak cross-Kerr nonlinearity, the demanded strength of nonlinearity can be compensated by using a probe coherent state with very large amplitude. For a realistic system, however, the intensity of the coherent beam cannot be boundlessly large because a laser beam with too strong intensity will bring about other effects in Kerr medium due to the effects of decoherence. Recently, as pointed out by Gea-Banacloche^49^, the large phase shifts via the giant Kerr effect with single-photon wave packets is impossible at present. A proper candidate for weak cross-Kerr nonlinearity should be atomic ensemble, and the fundamental problem with the cross-Kerr nonlinearity in atomic ensemble was discussed by Gea-Banacloche^49^, and He and Scherer^50^.

Besides the influence of cross-Kerr medium, the experiment feasibility of the present protocols also depends on the veracity of the X homodyne measurement. For the X homodyne measurement, we only consider the error chiefly coming from the overlap adjacent curves because of the fact that the coherent states of the probe beam with different phase shifts are not completely orthogonal. In fact, it is only one type of detection error in homodyne, other errors, such as the noises in detection, the reduced fidelity to the process in Eq. (2) due to multi-mode effect and decoherence, etc., also exist in a realistic implementation. Exploiting the appropriate measurement methods, the disadvantageous influence can be overcome or alleviated and the error probability will be decreased. In 2010, Wittmann *et al*. investigated quantum measurement strategies capable of discriminating two coherent states using a homodyne detector and a photon number resolving (PNR) detector^51^. In order to lower the error probability, the postselection strategy is applied to the measurement data of homodyne detector as well as a PNR detector. They indicated that the performance of the new displacement controlled PNR is better than homodyne receiver.

To summarize, we have proposed two quantum entanglement distribution protocols of a four-photon Dicke polarization entangled state over collective-noise channel with the assistance of the cross-Kerr nonlinearity. During transmission, the polarization DOF is easily disturbed over the collective-noise channel, we perform the entanglement conversion between polarization DOF and frequency DOF to eliminate the effect collective noise in the first quantum entanglement distribution protocol. After transmission, four receivers exploit the polarization independent WDMs to guide photons to different spatial modes according to their frequencies. At the same time, we fulfill another quantum entanglement distribution with the application of the spatial entanglement, which affords facilities for the experimental implementation and application because of releasing the polarization independent WDMs. These two protocols can be implemented deterministically in principle. By virtue of the availability of optical elements and techniques involved, we hope the present schemes for the distribution of entangled state in the polarization DOF can be experimentally implemented in the long-distance quantum communications.

## Additional Information

**How to cite this article**: Wang, M.-Y. *et al*. Deterministic distribution of four-photon Dicke state over an arbitrary collective-noise channel with cross-Kerr nonlinearity. *Sci. Rep.* **6**, 29853; doi: 10.1038/srep29853 (2016).

## Figures and Tables

**Table 1:** The distribution of the Dicke state with frequency entanglement.

|*ϕ*_*i*_〉	O.P.	D.P.	L.O. (A)	L.O. (B)	L.O. (C)	L.O. (D)
|*ϕ*_1_〉	*a*_1_*b*_1_*c*_1_*d*_1_	|*α*_1_*α*_2_*α*_3_*α*_4_|^2^	none	none	none	none
|*ϕ*_2_〉	*a*_1_*b*_1_*c*_1_*d*_2_	|*α*_1_*α*_2_*α*_3_*β*_4_|^2^	none	none	none	HWP
|*ϕ*_3_〉	*a*_1_*b*_1_*c*_2_*d*_1_	|*α*_1_*α*_2_*β*_3_*α*_4_|^2^	none	none	HWP	none
|*ϕ*_4_〉	*a*_1_*b*_1_*c*_2_*d*_2_	|*α*_1_*α*_2_*β*_3_*β*_4_|^2^	none	none	HWP	HWP
|*ϕ*_5_〉	*a*_1_*b*_2_*c*_1_*d*_1_	|*α*_1_*β*_2_*α*_3_*α*_4_|^2^	none	HWP	none	none
|*ϕ*_6_〉	*a*_1_*b*_2_*c*_1_*d*_2_	|*α*_1_*β*_2_*α*_3_*β*_4_|^2^	none	HWP	none	HWP
|*ϕ*_7_〉	*a*_1_*b*_2_*c*_2_*d*_1_	|*α*_1_*β*_2_*β*_3_*α*_4_|^2^	none	HWP	HWP	none
|*ϕ*_8_〉	*a*_1_*b*_2_*c*_2_*d*_2_	|*α*_1_*β*_2_*β*_3_*β*_4_|^2^	HWP	none	none	none
|*ϕ*_9_〉	*a*_2_*b*_1_*c*_1_*d*_1_	|*β*_1_*α*_2_*α*_3_*α*_4_|^2^	HWP	none	none	none
|*ϕ*_10_〉	*a*_2_*b*_1_*c*_1_*d*_2_	|*β*_1_*α*_2_*α*_3_*β*_4_|^2^	HWP	none	none	HWP
|*ϕ*_11_〉	*a*_2_*b*_1_*c*_2_*d*_1_	|*β*_1_*α*_2_*β*_3_*α*_4_|^2^	HWP	none	HWP	none
|*ϕ*_12_〉	*a*_2_*b*_1_*c*_2_*d*_2_	|*β*_1_*α*_2_*β*_3_*β*_4_|^2^	none	HWP	none	none
|*ϕ*_13_〉	*a*_2_*b*_2_*c*_1_*d*_1_	|*β*_1_*β*_2_*α*_3_*α*_4_|^2^	HWP	HWP	none	none
|*ϕ*_14_〉	*a*_2_*b*_2_*c*_1_*d*_2_	|*β*_1_*β*_2_*α*_3_*β*_4_|^2^	none	none	HWP	none
|*ϕ*_15_〉	*a*_2_*b*_2_*c*_2_*d*_1_	|*β*_1_*β*_2_*β*_3_*α*_4_|^2^	none	none	none	HWP
|*ϕ*_16_〉	*a*_2_*b*_2_*c*_2_*d*_2_	|*β*1*β*2*β*3*β*4|^2^	none	none	none	none

|*ϕ*_*i*_〉 denotes the collapsed polarization state after the X homodyne measurements, ‘O.P.’ represents the output mode where photon is detected, ‘D.P.’ denotes the corresponding detection probability, and ‘L.O.’ is the operations for photons.

**Table 2:** The distribution of the Dicke state with spatial entanglement.

*P*.*S*.	O.P.	D.P.	L.O. (A)	L.O. (B)	L.O. (C)	L.O. (D)
|*HHHH*〉	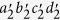	|*α*_1_*α*_2_*α*_3_*α*_4_|^2^	none	none	none	none
|*HHHV*〉	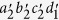	|*α*_1_*α*_2_*α*_3_*β*_4_|^2^	none	none	none	HWP
|*HHVH*〉	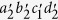	|*α*_1_*α*_2_*β*_3_*α*_4_|^2^	none	none	HWP	none
|*HHVV*〉	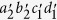	|*α*_1_*α*_2_*β*_3_*β*_4_|^2^	none	none	HWP	HWP
|*HVHH*〉	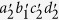	|*α*_1_*β*_2_*α*_3_*α*_4_|^2^	none	HWP	none	none
|*HVHV*〉	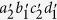	|*α*_1_*β*_2_*α*_3_*β*_4_|^2^	none	HWP	none	HWP
|*HVVH*〉	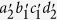	|*α*_1_*β*_2_*β*_3_*α*_4_|^2^	none	HWP	HWP	none
|*HVVV*〉	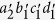	|*α*_1_*β*_2_*β*_3_*β*_4_|^2^	HWP	none	none	none
|*VHHH*〉	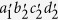	|*β*_1_*α*_2_*α*_3_*α*_4_|^2^	HWP	none	none	none
|*VHHV*〉	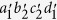	|*β*_1_*α*_2_*α*_3_*β*_4_|^2^	none	HWP	HWP	none
|*VHVH*〉	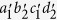	|*β*_1_*α*_2_*β*_3_*α*_4_|^2^	HWP	none	HWP	none
|*VHVV*〉	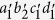	|*β*_1_*α*_2_*β*_3_*β*_4_|^2^	none	HWP	none	none
|*VVHH*〉	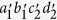	|*β*_1_*β*_2_*α*_3_*α*_4_|^2^	none	none	HWP	HWP
|*VVHV*〉	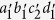	|*β*_1_*β*_2_*α*_3_*β*_4_|^2^	none	none	HWP	none
|*VVVH*〉	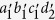	|*β*_1_*β*_2_*β*_3_*α*_4_|^2^	none	none	none	HWP
|*VVVV*〉	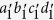	|*β*_1_*β*_2_*β*_3_*β*_4_|^2^	none	none	none	none

‘*P*.*S*.’ denotes the collapsed polarization state after X homodyne measurement, ‘ O.P.’ represents the output port where photon is detected, ‘D.P.’ denotes the corresponding detection probability, and ‘L.O.’ is the operations for photons.
